# Combined Effect of Per- and Polyfluoroalkyl Substances and Metals on Epigenetic Aging

**DOI:** 10.3390/toxics14050394

**Published:** 2026-05-04

**Authors:** Faustina Acquaah, Emmanuel Obeng-Gyasi

**Affiliations:** 1 Department of Built Environment, North Carolina A&T State University, Greensboro, NC 27411, USA; 2 Environmental Health and Disease Laboratory, North Carolina A&T State University, Greensboro, NC 27411, USA

**Keywords:** per- and polyfluoroalkyl substances (PFAS), heavy metals, cadmium, lead, epigenetic aging, DNA methylation, Bayesian kernel machine regression (BKMR), environmental mixtures, NHANES, biological aging

## Abstract

Environmental contaminants such as per- and polyfluoroalkyl substances (PFAS) and toxic metals have been implicated in biological aging, yet their combined effects remain poorly understood. This study evaluated the associations of PFAS, lead, and cadmium mixtures with multiple DNA methylation-based measures of epigenetic aging in a nationally representative sample of U.S. adults aged ≥ 50 years. Data were obtained from the 1999–2000 and 2001–2002 National Health and Nutrition Examination Survey (NHANES). The analytic sample included 1119 participants with available data on seven PFAS, blood lead, cadmium, and DNA methylation measures. Epigenetic aging outcomes included HannumAge, HorvathAge, SkinBloodAge, PhenoAge, GrimAge, and DunedinPoAm. Multivariable linear regression and Bayesian Kernel Machine Regression (BKMR) were used to assess individual and joint exposure–response relationships. Cadmium showed the most consistent positive associations with epigenetic aging measures, particularly for the second-generation clocks PhenoAge and GrimAge. Lead was positively associated with GrimAge, while PFAS showed more variable and generally weaker associations, with PFNA demonstrating the most consistent signal. Mixture analyses indicated that higher combined exposure levels were associated with higher DNA methylation age estimates, with stronger patterns observed for second-generation clocks. These findings suggest that combined exposure to PFAS, lead, and cadmium is associated with higher epigenetic aging in older U.S. adults, with cadmium emerging as a key contributor to the observed mixture effects. Evaluating environmental exposures as mixtures may provide important insight into how co-occurring contaminants jointly influence biological aging.

## 1. Introduction

Per- and polyfluoroalkyl substances (PFAS) are a diverse group of synthetic chemicals that are resistant to environmental degradation, resulting in their long-term persistence and bioaccumulation in human tissues. PFASs have been widely used in industrial applications and consumer products since the 1940s, including water-resistant textiles, greaseproof food packaging, personal care products, floor polishes, firefighting foams, and industrial surfactants [1,2]. Human exposure to PFAS is widespread and occurs through contaminated drinking water, food, consumer products, and inhalation of dust and air [3,4,5]. In addition to PFAS, exposure to toxic metals such as lead (Pb) and cadmium (Cd) remains a significant public health concern due to their widespread distribution, persistence, and bioaccumulation. Lead exposure occurs through sources such as lead-based paints, contaminated soil, drinking water transported through lead pipes, and specific consumer products [6,7,8]. Cadmium exposure is commonly linked to cigarette smoke, contaminated food, industrial emissions, and occupational settings such as battery manufacturing and metal smelting [9,10,11]. PFAS and metals have been associated with adverse health outcomes, including metabolic dysfunction, immune dysregulation, cardiovascular disease, and cancer [12,13]. Increasingly, environmental exposures are being recognized as important contributors to biological aging, a process characterized by progressive physiological decline and increased susceptibility to disease and mortality [14]. Age can be characterized using chronological age, which reflects time lived, and biological age, which captures variation in cellular and organ-system integrity among individuals of the same chronological age [15,16]. 

Biological aging can be estimated using DNA methylation-based measures of epigenetic aging, which capture molecular changes associated with aging processes [16]. Epigenetics involves chemical modifications to DNA or its associated proteins that regulate gene expression without changes to the DNA sequence, and these processes are influenced by both genetic and environmental factors [17,18]. Disruptions to DNA methylation may occur in response to toxic exposures and contribute to increased risk of cardiovascular, metabolic, and cancer-related diseases [19,20]. Epigenetic clocks, constructed from DNA methylation patterns at specific CpG sites, provide estimates of biological age and have become strong predictors of morbidity and mortality [21,22,23]. First-generation epigenetic clocks, including Hannum, Horvath, and Skin & Blood, were developed to estimate chronological age [24,25,26], whereas second- and third-generation clocks capture broader aspects of biological aging and health risk. These include PhenoAge, which integrates clinical biomarkers to predict morbidity and mortality [27]; GrimAge, a strong predictor of lifespan and disease risk [28,29]; and DunedinPoAm, which quantifies the pace of aging and is associated with healthspan and functional decline [30]. Prior studies indicate that PFAS and heavy metals can alter DNA methylation at genes involved in inflammation, detoxification, and metabolic regulation [31,32]. PFAS-related epigenome-wide association studies have identified methylation changes across multiple life stages, and some evidence suggests that these alterations may mediate cardiometabolic outcomes [33,34,35]. Mechanistic evidence also implicates PFAS, lead, and cadmium in oxidative stress, chronic inflammation, endocrine disruption, mitochondrial dysfunction, and dysregulation of DNA methylation pathways [36,37,38,39].

Despite this evidence, important gaps remain. Several studies have evaluated metals and PFAS separately using individual-exposure and mixture-based approaches [40,41,42]. Additionally, one study evaluated metals alongside other environmental chemicals, but PFASs were not included [43]. To our knowledge, no prior studies have jointly evaluated PFAS and metals within a unified mixture framework, limiting understanding of how these co-occurring contaminants may influence biological aging. Therefore, the objective of this study was to evaluate the combined effects of PFAS, lead, and cadmium on multiple epigenetic clocks in older U.S. adults. This study extends prior research by jointly examining PFAS and metals within a mixture context and applying Bayesian Kernel Machine Regression to capture potential nonlinear and joint exposure–response relationships.

## 2. Materials and Methods

### 2.1. Study Population

This study analyzed data from the 1999–2000 and 2001–2002 cycles of the National Health and Nutrition Examination Survey (NHANES), a nationally representative, cross-sectional survey conducted by the National Center for Health Statistics (NCHS) of the U.S. Centers for Disease Control and Prevention (CDC) to assess the health and nutritional status of the non-institutionalized U.S. population. NHANES employs a complex, multistage probability sampling design and collects comprehensive demographic, physical examination, and laboratory data. All participants provided written informed consent, and the NCHS Research Ethics Review Board approved the study protocols [44].

**Figure 1:**
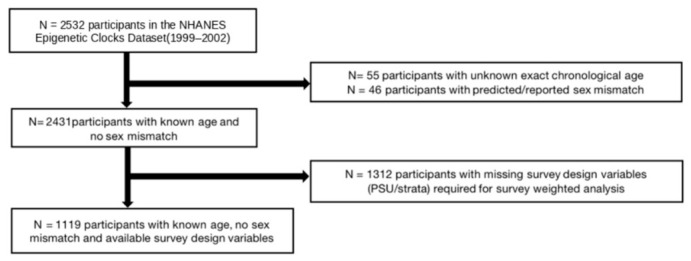
Flowchart depicting the sample size for the study.

This analytic subsample included 2532 adults aged ≥ 50 years with available data on PFSA, PFNA, PFHS, PFOA, PFOS, EPAH, MPAH, lead, cadmium, and blood DNA methylation. In NHANES, age is top-coded at 85 years to protect participant confidentiality; therefore, participants with top-coded age values (≥85 years; *N* = 55) were excluded because their exact chronological ages are unavailable, which may lead to misclassification of age in age-dependent analyses. Participants whose DNA methylation-predicted sex did not match their self-reported sex were excluded (*N* = 46). Participants missing NHANES survey design variables (primary sampling unit or stratum identifiers) were excluded, as these variables are required to specify the complex survey design and obtain valid variance estimates in survey-weighted analyses. The final analytic sample consisted of 1119 participants. Missing covariate data within the analytic sample were addressed using multiple imputation. The derivation of the analytic sample, including all exclusion steps and corresponding sample sizes, is summarized in Figure 1.

#### 2.1.1. PFAS Biomarker Measurement

11 serum PFAS concentrations including perfluorooctane sulfonamide (PFOSA), 2-(N-ethyl-perfluorooctane sulfonamido) acetic acid (Et-PFOSA-AcOH), 2-(N-methyl-perfluorooctane sulfonamido) acetic acid (Me-PFOSA-AcOH), perfluorohexane sulfonic acid (PFHS), PFOS, PFOA, perfluorohexanoic acid (PFHxA), perfluorononanoic acid (PFNA), perfluorodecanoic acid (PFDeA), perfluoroundecanoic acid (PFUA), and perfluorododecanoic acid (PFDoA) were measured using online solid-phase extraction coupled with high-performance liquid chromatography–tandem mass spectrometry (HPLC–MS/MS). Serum samples were diluted with 0.1 M formic acid and analyzed using a column-switching system with a C18 extraction column followed by separation on a C8 analytical column. Analytes were detected by negative-ion TurboIonSpray electrospray ionization-tandem mass spectrometry. Quantification was based on isotope-labeled internal standards (^18^O_2_-PFOSA, ^18^O_2_-PFOS, and ^13^C_2_-PFOA), with calibration standards prepared in calf serum to account for matrix effects. Method performance, including limits of detection, accuracy, and precision, was assessed using spiked serum samples, and PFOS concentrations were corrected for endogenous levels in calf serum [45,46]. PFAS concentrations below the lower limit of detection (LOD) were replaced with LOD/√2. Compounds with detection frequencies below 50% in the study population (PFDeA, PFDoA, PFHxA, PFUA) were excluded from the analysis.

#### 2.1.2. Lead and Cadmium Measurement

Lead and cadmium concentrations in blood were measured using simultaneous multi-element atomic absorption spectrometry. Absorbance was measured at 283.3 nm for lead and 228.8 nm for cadmium using electrodeless discharge or hollow cathode lamps. Whole blood samples, quality control materials, and calibration standards were diluted with a matrix modifier containing nitric acid, Triton X-100, and ammonium phosphate, and analyzed on a PerkinElmer SIMAA 6000 spectrometer with Zeeman background correction (PerkinElmer, Norwalk, CT, USA) [47].

#### 2.1.3. DNA Methylation Measurements and Epigenetic Aging

DNA was extracted from whole blood, and specimens were stored at −80 °C. The DNAm assay was performed at Duke University. Bisulfite conversion of DNA was carried out using the manufacturer’s recommendations. Five hundred nanograms of DNA were bisulfite-treated using a Zymo EZ DNA Methylation kit (cat# D5001, Zymo Research, Irvine, CA, USA) under PCR conditions for Illumina’s Infinium Methylation assay. Data were produced on the Illumina Infinium MethylationEPIC BeadChip v1.0 (cat# WG317-1001, Illumina, San Diego, CA, USA), and chips were imaged using the Illumina iScan system. For the NHANES biomarker release, IDAT files were processed in R (version 4.3.1) within the RStudio environment. The NHANES DNAm biomarker pipeline included preprocessing of IDAT files into methylated and unmethylated signals, control-probe-based background subtraction and color correction, sample outlier removal, biomarker-specific imputation, and normalization. For HorvathAge, HannumAge, SkinBloodAge, PhenoAge, and GrimAge, missing CpG values were imputed using the Horvath gold-standard reference data set; for DunedinPoAm, mean CpG values from the NHANES dataset were used for imputation. Probe-type bias was addressed using beta mixture quantile normalization (BMIQ), with a modified Horvath-based normalization approach used for HorvathAge, HannumAge, SkinBloodAge, PhenoAge, and GrimAge. Published biomarker coefficients were then applied to the corresponding CpG subsets to generate epigenetic biomarker scores. Cell-type proportions were estimated using the IDOL probe set and the FlowSorted.Blood.EPIC reference dataset, and sample mismatch assessment included evaluation of age deviation, cell-type deviation, and XY chromosomal ploidy. Detailed probe-level quality-control and filtering procedures for the NHANES DNA methylation data products, including handling of poorly performing probes, polymorphic probes, cross-hybridizing probes, and XY chromosome probes, are described in the NHANES DNA Methylation Array and Epigenetic Biomarkers documentation [48].

Analyses included the Hannum, Horvath pan-tissue, Skin & Blood, PhenoAge, GrimAge, and DunedinPoAm epigenetic clocks. Raw epigenetic clock values were modeled as continuous outcomes for all analyses. In regression and mixture models, chronological age was included as a covariate to account for age-related differences in DNA methylation age estimates. Residual-based epigenetic age acceleration (EAA) measures were not derived. This modeling framework evaluates whether environmental exposures are associated with higher biological age estimates among individuals of the same chronological age.

DunedinPoAm, which quantifies the pace of biological aging and is less dependent on chronological age than traditional clocks, was also modeled with chronological age adjustment to ensure analytic consistency across all clocks, comparability across models, and to account for any residual age-related variation.

### 2.2. Variables and Covariates

The primary outcome variable was epigenetic aging, a biomarker of biological aging. Predictor variables included environmental chemical exposures, specifically 7 PFAS (PFSA, PFNA, PFHS, PFOA, PFOS, EPAH, MPAH), as well as lead and cadmium. Statistical models adjusted for demographic covariates (age, sex, and race/ethnicity), socioeconomic indicators (education and household income), and body mass index (BMI). Age and BMI were modeled as continuous variables, while sex, race/ethnicity, education, and household income were included as categorical variables based on standard NHANES classifications.

### 2.3. Statistical Analysis

#### 2.3.1. Descriptive Statistics and Correlation Analysis

Descriptive statistics were used to describe participant characteristics and the distribution of study variables. Continuous variables were summarized using means and standard deviations, while categorical variables were described using counts and percentages. Variables summarized included age, sex, race/ethnicity, education, income, and BMI.

Following descriptive analysis, Spearman’s rank correlation coefficients were calculated to assess pairwise associations among PFAS, lead, cadmium, and epigenetic aging measures. Spearman’s rank correlation assesses monotonic relationships based on ranked values rather than raw measurements, making it less sensitive to outliers and to distributional assumptions.

The Spearman rank correlation is defined as:

$$r_{s}=1-\frac{6\sum d_{i}^{2}}{n(n^{2}-1)}$$

where $d_{i}$ is the difference between the ranks of the *i*th pair, and n is the sample size. Values of $r_{s}$ range from −1 to +1, with positive values reflecting a positive association, negative values reflecting a negative association, and values near zero indicating no association.

#### 2.3.2. Linear Regression Analysis

Multivariable linear regression analyses were conducted to evaluate associations between individual metals, PFAS, and epigenetic aging measures. Epigenetic age estimates derived from the Hannum, Horvath pan-tissue, Skin & Blood, PhenoAge, and GrimAge clocks, as well as DunedinPoAm, a measure of the pace of biological aging, were examined. All models included lead, cadmium, EPAH, MPAH, PFHS, PFNA, PFOA, PFOS, and PFSA as predictors. Models for epigenetic clock estimates were adjusted for age, sex, race/ethnicity, education, income, and body mass index. The linear regression equation was as follows:

$$Y_{i}=\beta_{0}+\beta_{1}X_{i}+\beta_{2}Z_{i}+\varepsilon_{i}$$

where $Y_{i}$ denotes the epigenetic aging outcome for participant *i*, $X_{i}$ represents exposure variables, $Z_{i}$ denotes covariates, β coefficients represent estimated associations between predictors and the outcome, and $\varepsilon_{i}$ ~ N (0, $\sigma^{2}$) is the random error term.

#### 2.3.3. Bayesian Kernel Machine Regression (BKMR)

Bayesian Kernel Machine Regression (BKMR) was employed to evaluate the joint and potentially non-linear effects of PFAS, lead, and cadmium on epigenetic aging outcomes. This approach is well-suited for mixture analyses because it flexibly models complex exposure–response relationships while accounting for interactions and correlations among exposures [49,50].

The BKMR model was specified as:

$$Y_{i}=h\left(Z_{i}\right)+X_{i}^{T}\beta +\varepsilon_{i}$$

where $Y_{i}$ represents the health outcome, *h* is a flexible kernel function, $Z_{i}={\left(Z_{i1}\ldots ,Z_{iM}\right)}^{T}$ and denotes the vector of exposure variables. The term $X_{i}$ represents a vector of covariates, β represents the corresponding regression coefficients, and $\varepsilon_{i}$ an error term is assumed to follow a normal distribution.

Posterior inference was performed using a single Markov chain Monte Carlo (MCMC) chain with 5000 iterations and variable selection enabled, with the first 1000 iterations discarded as burn-in. Convergence was assessed by visual inspection of trace plots. No thinning was applied. A Gaussian kernel function was used to capture potentially non-linear and non-additive relationships between exposures and outcomes, and variable selection was implemented within the BKMR framework. Exposure variables were analyzed on their original scale without log transformation. To improve numerical stability and ensure comparability across exposures, all variables were mean-centered and standardized before model fitting. High-dimensional exposure–response functions were estimated to characterize the overall mixture effect, as well as the individual exposure–response relationships for each chemical while holding all other exposures at their median values. These functions allowed visualization of how epigenetic aging outcomes varied across the exposure distribution and facilitated interpretation of both cumulative and individual effects within the mixture.

To assess the relative importance of individual exposures within the mixture, posterior inclusion probabilities (PIPs) were estimated. PIPs quantify the probability that a given exposure contributes meaningfully to the mixture effect while accounting for correlations and interactions among chemicals. Higher PIP values indicate greater importance of an exposure in predicting the outcome. This approach enabled the identification of key contributors within the PFAS and metal mixture influencing epigenetic aging.

Models adjusted for age, sex, race/ethnicity, educational attainment, annual household income, and body mass index (BMI).

All BKMR, multivariable linear regression, and Spearman correlation analyses were conducted using R software (version 4.2.3; R Foundation for Statistical Computing, Vienna, Austria). Descriptive statistics were performed using Stata SE version 19.5 (StataCorp, College Station, TX, USA), with a significance level of 0.05 for non-Bayesian analyses. Summary statistics incorporated subsample weights and accounted for the complex design of the NHANES survey.

#### 2.3.4. Missing Data Handling

Multiple Imputation by Chained Equations (MICE) was employed to address missing data, with 28 imputations to reflect an overall missingness of approximately 28% in the analytic dataset [51]. Before imputation, missing values were observed for PFAS biomarkers (*n* = 816 across all seven PFAS), lead (*n* = 1), cadmium (*n* = 1), BMI (*n* = 15), and household income (*n* = 184).

The imputation model included all analytic variables used in the study, including exposures, epigenetic aging outcomes, and covariates. Imputation was performed using the mice package in R, with variable-specific methods selected automatically based on data type. Continuous variables were imputed using predictive mean matching, which helps maintain plausible values and the overall distribution of the observed data [52].

The MICE approach assumes data are missing at random (MAR) such that the probability of missingness depends on observed variables but not on unobserved values [51]. This assumption was considered reasonable given the availability of observed covariates related to missingness, which were included in the imputation models. This approach reduced potential bias associated with missing data and improved the efficiency and robustness of subsequent analyses. Completed data generated from the multiple imputation procedure were used in the subsequent regression and mixture modeling analyses.

No formal multiple comparison correction was applied. Given the exploratory and hypothesis-generating nature of the mixture and multi-outcome analyses, findings were interpreted cautiously, with emphasis placed on consistency of patterns across models and epigenetic aging measures rather than isolated statistical significance.

## 3. Results

### 3.1. Descriptive Statistics

Descriptive statistics for the continuous variables, including age, body mass index (BMI), and environmental exposures, are presented in Table 1. In the survey-weighted population, participants had a mean age of 63.2 years, reflecting an older adult population, and a mean BMI of 28.8 kg/m^2^. Mean concentrations of lead and cadmium were 2.71 (SE = 0.12) and 0.70 (SE = 0.04) µg/dL, respectively. Among PFAS, PFOS showed the highest mean concentration of 38.25 (SE = 2.63), followed by PFOA (6.14, SE = 0.52) and PFHS (2.96, SE = 0.22). EPAH (0.81, SE = 0.08), MPAH (0.90, SE = 0.10), PFNA (0.81, SE = 0.07) and PFSA (0.50, SE = 0.03) had relatively lower mean concentrations.

The distributions of categorical variables, including sex, race/ethnicity, income, and educational attainment, are summarized in Table 2. The weighted sample was nearly evenly distributed by sex, with 55.0% women and 45.0% men. Most participants identified as Non-Hispanic White (75.9%), followed by Non-Hispanic Black (8.9%), Other Hispanic (7.4%), Mexican American (4.3%), and individuals of other or multiracial backgrounds (3.5%). Educational attainment varied across the population, with 12.0% reporting less than a 9th-grade education, 20.7% completing grades 9–11 without a diploma, 27.3% completing high school or a GED, 22.2% reporting some college or an associate degree, and 17.9% holding a college degree or higher. Annual household income varied across categories, with 28.3% reporting income below USD 20,000, 21.8% reporting USD 20,000–USD 34,999, 19.8% reporting USD 35,000–USD 54,999, and 30.2% reporting income above USD 55,000.

Table 3 summarizes results from multivariable linear regression models examining associations between individual metal and PFAS exposures and six epigenetic aging measures: HannumAge, HorvathAge, SkinBloodAge, PhenoAge, GrimAge, and DunedinPoAm. Regression coefficients represent the estimated change in each epigenetic aging measure per one-unit increase in exposure, conditional on covariates.

Cadmium showed the strongest and most consistent statistically significant positive associations with epigenetic aging across multiple clocks. Effects were modest for first-generation clocks but stronger for second-generation measures, particularly PhenoAge and GrimAge.

Lead and PFNA were also positively associated with aging, notably with GrimAge. PFNA showed additional positive but non-significant associations with other clocks.

Other PFAS displayed heterogeneous patterns. PFHS and PFOA were generally positively associated, whereas PFSA and PFOS were mostly inversely related to epigenetic aging. EPAH and MPAH showed variable associations, with EPAH more often inversely related to second-generation clocks.

### 3.2. Spearman Correlation Matrix Analysis

**Figure 2:**
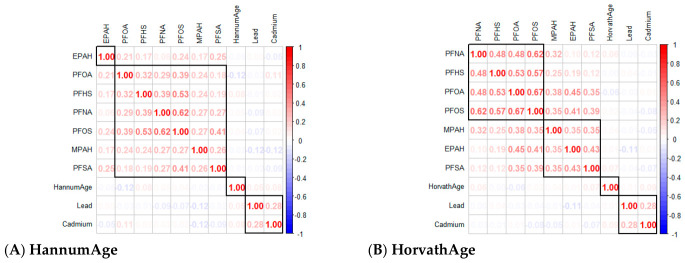

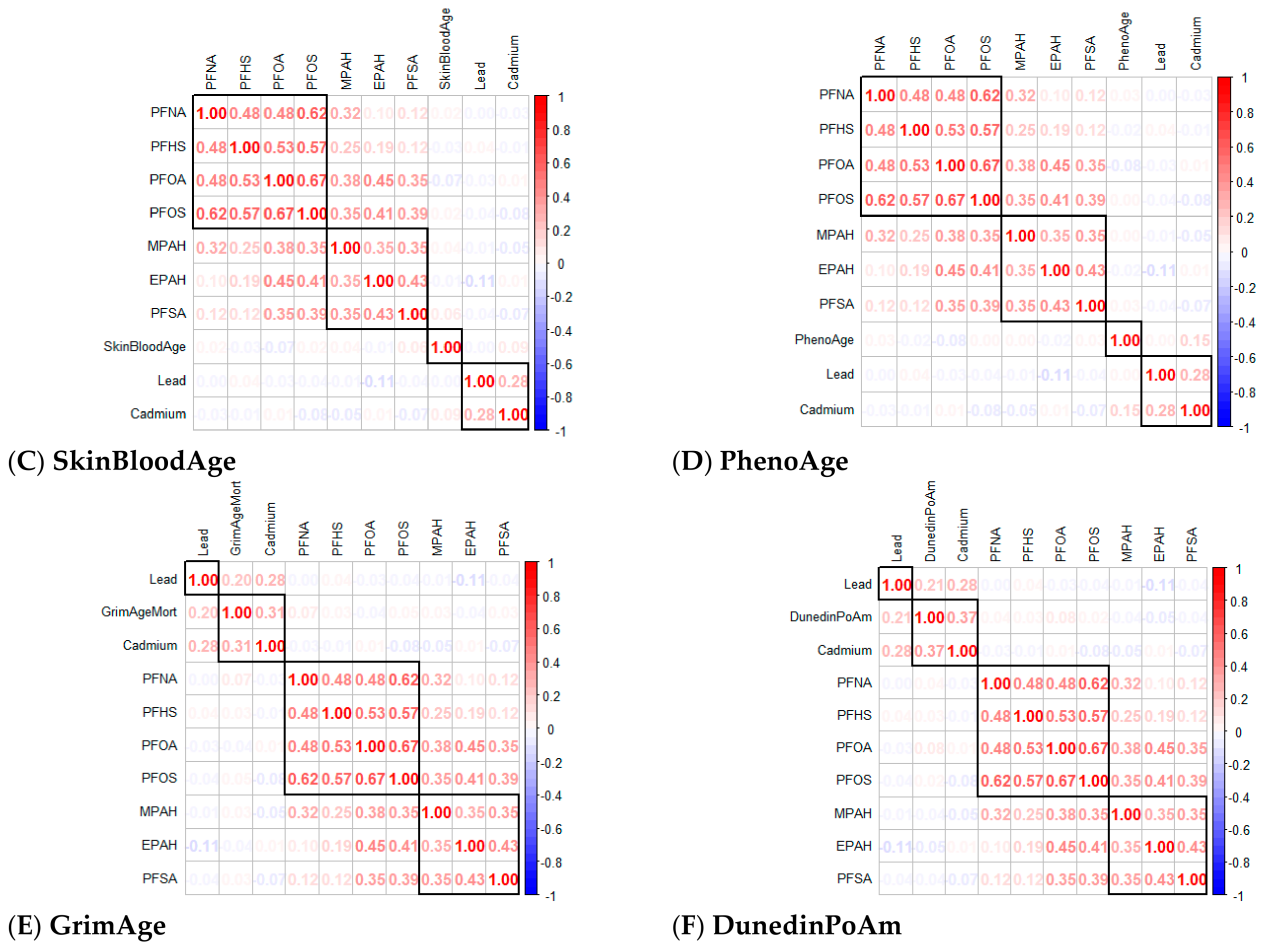
Spearman correlation matrices showing pairwise correlations among PFAS, lead, cadmium, and epigenetic aging measures. Color indicates the direction and strength of correlations (red = positive, blue = negative), with darker shades representing stronger relationships.

Figure 2A–F presents Spearman correlation matrices for each epigenetic aging measure in relation to PFAS biomarkers and metals. PFAS compounds were moderately to strongly intercorrelated, particularly PFOS, PFOA, PFNA, and PFHS, indicating shared exposure sources. Correlations between metals were weak, with lead and cadmium showing only a modest association.

Correlations between environmental exposures and epigenetic aging measures were generally weak for first-generation clocks (HannumAge, HorvathAge, and SkinBloodAge). PFAS biomarkers showed weak correlations across all clocks. Second-generation measures (PhenoAge and GrimAge) showed modest positive correlations with cadmium and lead. DunedinPoAm showed similar modest positive correlations with metals but remained weakly correlated with PFAS biomarkers.

### 3.3. Bayesian Kernel Machine Regression Results

Within the BKMR framework, posterior inclusion probabilities and univariate, bivariate, overall, single-variable, and single-variable interactive exposure–response relationships were evaluated to characterize the joint and individual contributions of PFAS and metal exposures to epigenetic aging outcomes. The results of these analyses are detailed in the subsequent sections.

#### 3.3.1. Posterior Inclusion Probability

Table 4 presents posterior inclusion probabilities, which quantify the relative importance of individual PFAS and metal exposures in explaining variability in epigenetic aging outcomes within the Bayesian kernel machine regression framework. Higher posterior inclusion probabilities indicate a greater contribution of a given exposure to the overall mixture effect.

Posterior inclusion probabilities demonstrated clear outcome-specific patterns. Cadmium showed high to very high posterior inclusion probabilities for HannumAge, PhenoAge, GrimAge, and DunedinPoAm, indicating a dominant contribution among the modeled exposures for these outcomes. In contrast, HorvathAge exhibited uniformly low posterior inclusion probabilities across all exposures.

SkinBloodAge displayed a more distributed mixture profile, with EPAH showing the highest posterior inclusion probability, followed by cadmium and smaller contributions from several PFAS. For PhenoAge, multiple PFAS and lead exhibited moderate posterior inclusion probabilities, whereas PFAS contributions to GrimAge and DunedinPoAm were minimal.

#### 3.3.2. Univariate, Bivariate, Overall, Single-Variable, and Single-Variable Interactive Effects

**Figure 3:**
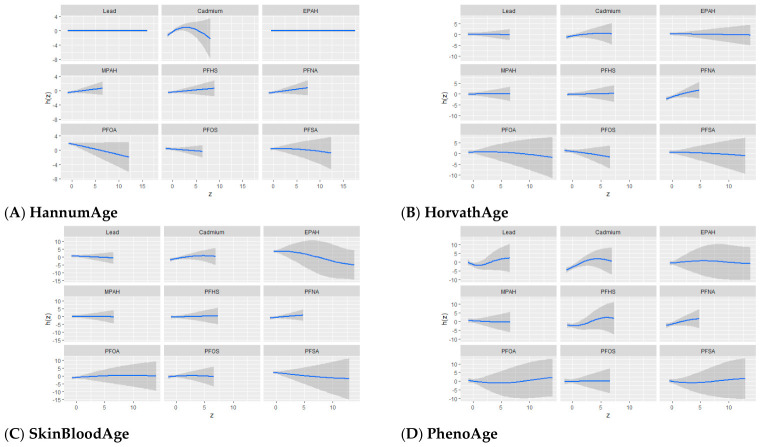

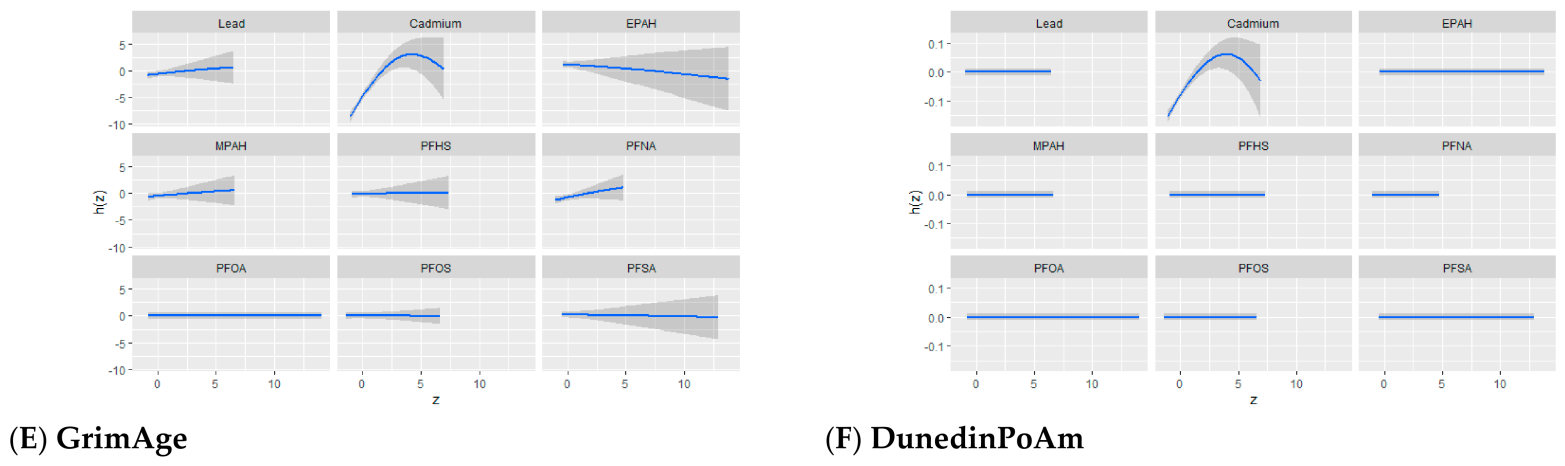
Univariate exposure–response functions showing the association between individual PFAS and metal exposures and epigenetic aging outcomes. Solid lines represent estimated effects, and grey areas indicate 95% credible intervals, with all other exposures held at their median values. Models were adjusted for age, sex, race/ethnicity, education, income, and BMI.

**Figure 4:**
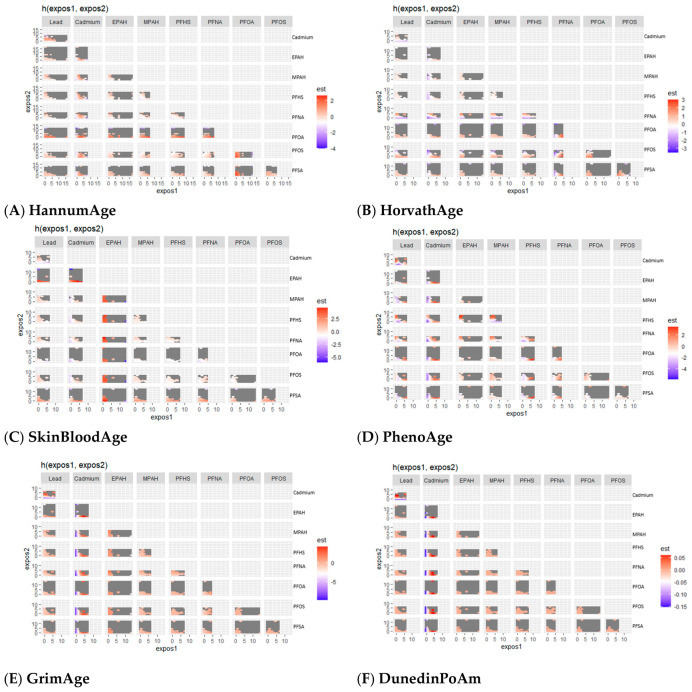
Bivariate exposure–response functions showing joint effects of PFAS and metal exposures on epigenetic aging outcomes. Each panel shows how the effect of one exposure changes across its range at different levels of a second exposure. Colors indicate the direction and strength of associations (red = positive, blue = negative), with darker shades representing stronger effects. Models were adjusted for age, sex, race/ethnicity, education, income, and BMI.

**Figure 5:**
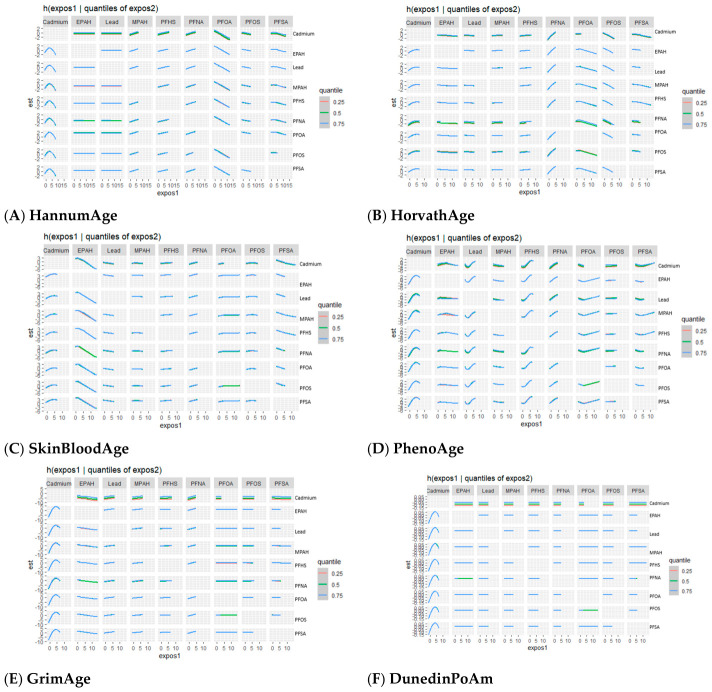
Quantile-based bivariate exposure–response functions showing how the association between one exposure and epigenetic aging varies across the 0.25, 0.50, and 0.75 quantiles of a second exposure. The x-axis represents the first exposure, and the y-axis the estimated effect. Colored lines (red, green, and blue) correspond to the 0.25, 0.5, and 0.75 quantiles of the second exposure, with other exposures held at their median values. Models were adjusted for age, sex, race/ethnicity, education, income, and BMI.

**Figure 6:**
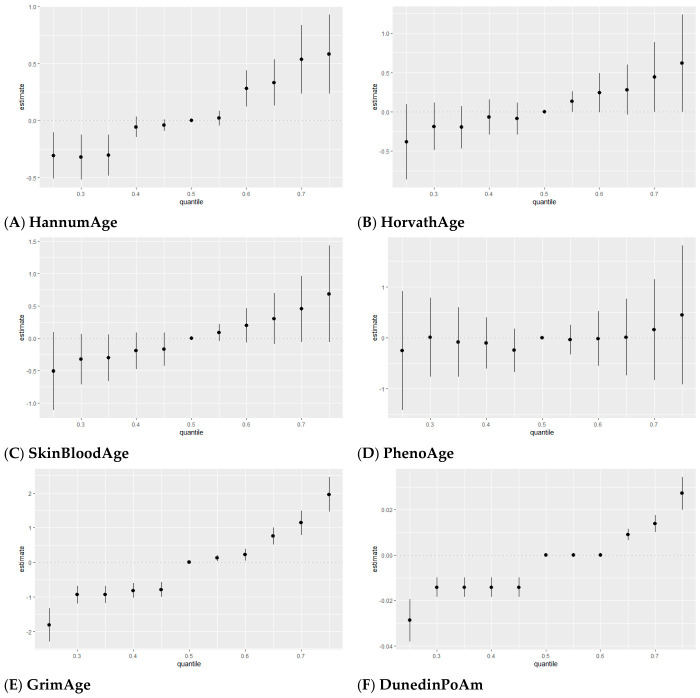
Overall mixture effect of combined PFAS and metal exposures on epigenetic aging outcomes. The plot shows the estimated change in epigenetic aging as all exposures increase from the 25th to 75th percentile, relative to the 50th percentile. Points represent estimated effects, and vertical lines indicate 95% credible intervals. Models were adjusted for age, sex, race/ethnicity, education, income, and BMI.

**Figure 7:**
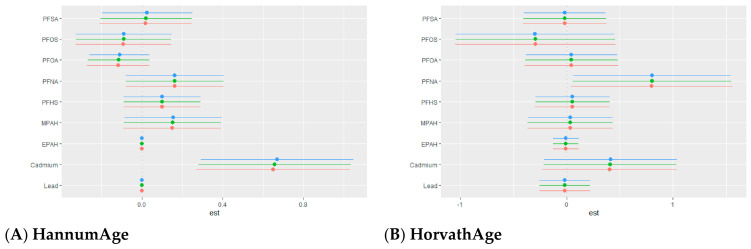

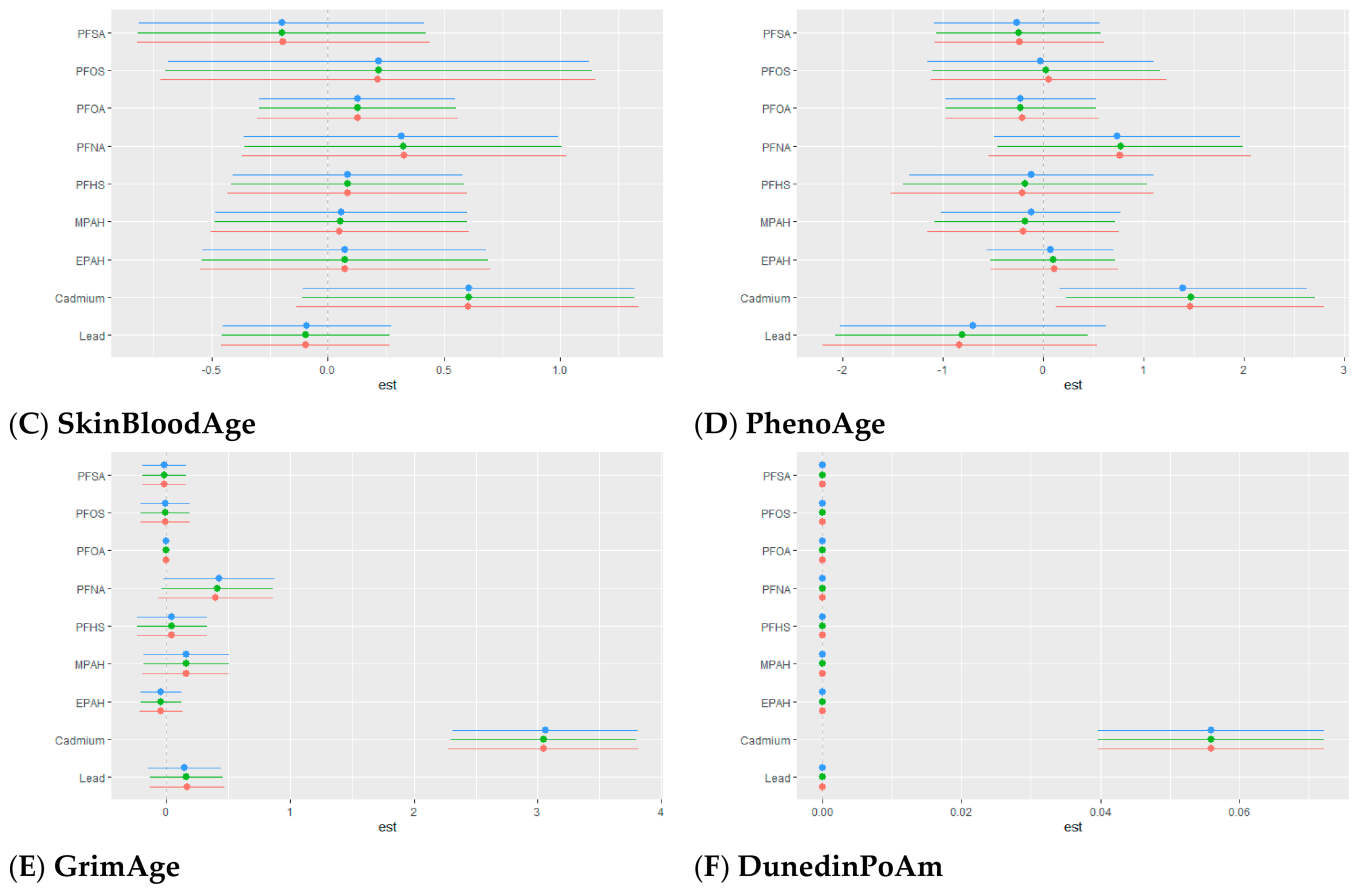
Single-exposure effects of individual PFAS and metals on epigenetic aging outcomes. Estimates represent the change in epigenetic aging associated with increasing each exposure from the 25th to the 75th percentile, while holding all other exposures at fixed levels (0.25 (red), 0.50 (green), or 0.75 (blue)). Points show estimated effects, and horizontal bars indicate 95% credible intervals. Models were adjusted for age, sex, race/ethnicity, education, income, and BMI.

**Figure 8:**
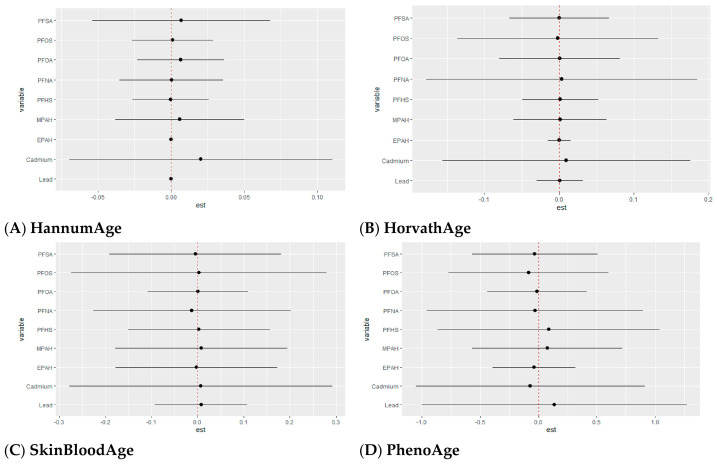

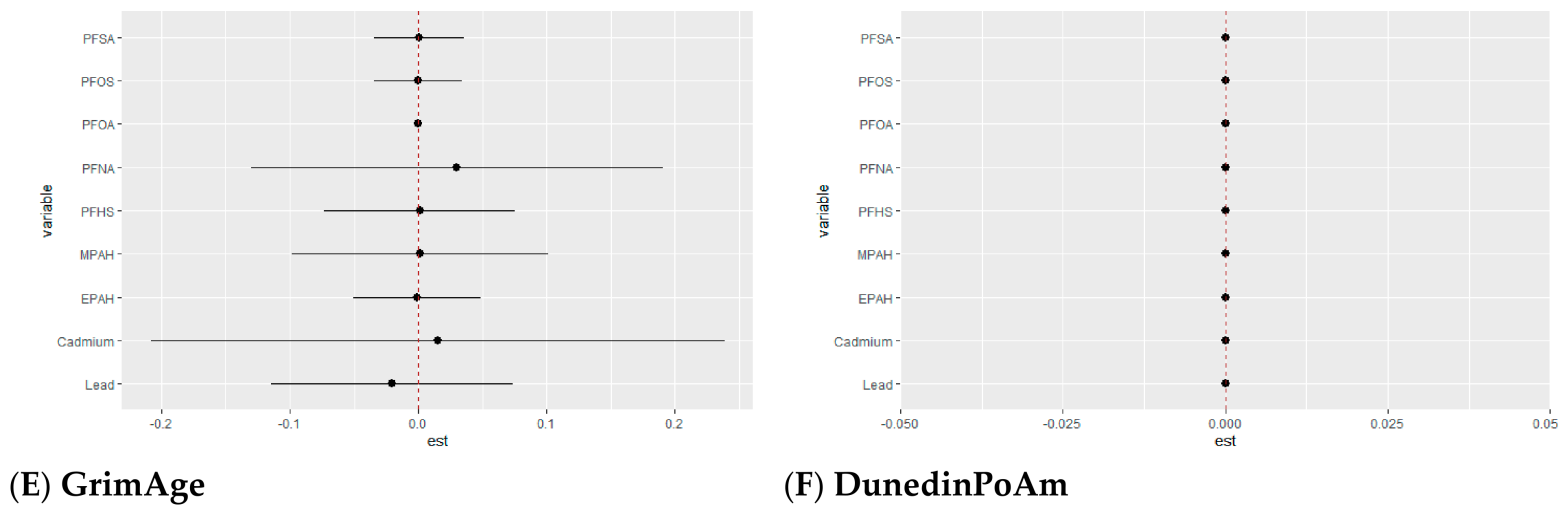
Single-exposure interaction effects of PFAS and metals on epigenetic aging outcomes. Estimates represent the change in the exposure–outcome association when other exposures are fixed at higher versus lower levels. Points indicate posterior mean estimates, and horizontal lines represent 95% credible intervals. Models were adjusted for age, sex, race/ethnicity, education, income, and BMI.

In this section, we present results from the BKMR analyses examining associations between environmental exposures and epigenetic aging outcomes, including HannumAge, HorvathAge, SkinBloodAge, PhenoAge, GrimAge, and DunedinPoAm. For each exposure–response function, Figure 3, Figure 4, Figure 5, Figure 6, Figure 7 and Figure 8 present six panels corresponding to the six epigenetic aging outcomes, allowing direct comparison of exposure-specific patterns across clocks. These visualizations characterize univariate and bivariate exposure–response relationships, assess potential interactions within the exposure mixture, and summarize the overall and quantile-specific contributions of individual exposures.

##### Univariate Exposure–Response Function of Exposures and Epigenetic Aging Measures

Figure 3 presents univariate exposure–response functions for associations between individual exposures and epigenetic aging measures. The exposure–response functions provide insight into the direction and magnitude of the relationships between each exposure and epigenetic aging, while other exposures are held at their median values. The shaded areas around each curve represent the 95% credible intervals, indicating the level of uncertainty around the estimated associations. In this figure, “z” represents the value of a specific exposure, and “h(z)” represents the predicted outcome based on that exposure.

Cadmium showed the most pronounced exposure–response patterns across outcomes, with evidence of nonlinearity characterized by steeper increases at lower to moderate concentrations and attenuation at higher levels, particularly for HannumAge, PhenoAge, GrimAge, and DunedinPoAm. In contrast, lead and most PFAS exhibited largely flat or weak relationships. Among PFAS, PFNA showed the most consistent positive associations across several clocks, while PFHS showed small positive associations for selected outcomes. EPAH demonstrated an inverse pattern for SkinBloodAge. Overall, PFAS associations were modest and accompanied by wider credible intervals, especially at higher exposure levels.

##### Bivariate Exposure–Response Functions Illustrating the Joint Association of PFAS/Metal Exposures with Epigenetic Aging Measures

Figure 4 presents bivariate exposure–response functions, illustrating the joint associations between paired PFAS and metal exposures and epigenetic aging measures, with other exposures held at their median values.

Joint exposure patterns were heterogeneous and outcome-specific. For first-generation clocks, associations were generally modest. HannumAge showed localized positive associations for cadmium at moderate levels when paired with lead and selected PFAS. HorvathAge showed limited joint sensitivity, with modest positive associations observed for higher PFNA and moderate cadmium levels. SkinBloodAge exhibited a broader response, with positive associations observed for EPAH and PFSA at lower to moderate exposure levels.

Second-generation measures showed more structured joint patterns. PhenoAge exhibited positive associations at higher lead and cadmium levels under low to moderate co-exposure conditions. GrimAge and DunedinPoAm showed nonlinear patterns for cadmium, with inverse associations at lower concentrations and positive associations at moderate to high concentrations, when co-exposures were low. Across outcomes, joint PFAS effects were modest and accompanied by greater uncertainty.

##### Quantile-Based Bivariate Exposure–Response Functions

Figure 5 presents quantile-based bivariate exposure–response functions estimated using BKMR, illustrating how the association between one exposure and epigenetic aging changes across different levels of a second exposure. Each panel represents a pair of exposures, allowing assessment of potential interactions and how the effect of one exposure strengthens, weakens, or remains stable across levels of a co-exposure.

Across outcomes, quantile-specific joint associations were generally modest, with limited separation across quantiles for most exposure pairs, indicating largely stable relationships and weak interaction overall. Cadmium showed the most consistent evidence of interaction, with its association varying across quantiles of co-exposures. Specifically, as cadmium levels increased, its association with epigenetic aging strengthened at lower to moderate concentrations and attenuated at higher levels, consistent with inverted U-shaped patterns observed for HannumAge, PhenoAge, GrimAge, and DunedinPoAm.

Most PFAS pairs showed minimal interaction, with largely overlapping curves across quantiles, indicating stable associations. Lead showed mostly weak, flat patterns across outcomes, although a modest nonlinear (U-shaped) association was observed for PhenoAge, with limited separation across quantiles.

For first-generation clocks, interactions were weak across exposure pairs, although cadmium showed nonlinear exposure–response patterns. Among second-generation measures, PhenoAge showed the clearest nonlinear patterns across several exposures, driven primarily by cadmium, with some curvature also observed for selected PFAS and lead. In contrast, associations for GrimAge and DunedinPoAm were mostly flat, with little change across quantiles.

##### Overall Exposure Effect of Multiple Pollutants on Epigenetic Aging Outcomes Across Quantiles

Figure 6 shows how epigenetic aging changes as PFAS and metal exposures increase together across their distribution. The x-axis represents exposure percentiles, and the y-axis shows the estimated change in epigenetic aging relative to the median, allowing assessment of whether the overall mixture effect strengthens, weakens, or remains stable at higher exposure levels. For first-generation clocks, effects were modest. HannumAge and HorvathAge showed lower estimates at lower exposure levels, with gradual increases at higher levels. SkinBloodAge showed a clearer, more consistent increase across the exposure range.

For second and third-generation measures, patterns differed across outcomes. GrimAge showed the strongest response, with a clear shift from negative estimates at lower exposure levels to positive estimates at higher levels. PhenoAge showed small changes across the exposure range, with estimates remaining close to the null and greater uncertainty. DunedinPoAm showed a mild nonlinear pattern, with lower estimates at lower exposure levels and higher estimates at higher levels.

##### Single-Variable Effects of Metals and PFAS on Epigenetic Aging

Figure 7 shows the single-exposure effects of PFAS and metals on epigenetic aging across different mixture levels, allowing assessment of how each exposure independently contributes to aging while accounting for other exposures. Effect estimates are shown on the x-axis, with horizontal lines indicating 95% credible intervals. Colors represent different mixture levels (0.25, 0.50, and 0.75), showing how these associations vary across the exposure distribution.

Across epigenetic clocks, single-exposure analyses revealed both shared and clock-specific patterns. Cadmium was the most consistent exposure across outcomes, showing positive associations with all epigenetic clocks, with variation in magnitude.

Among first-generation clocks, HannumAge showed positive associations with cadmium, while most PFAS exhibited weak or near-null effects. For HorvathAge, PFNA showed the largest positive association, followed by cadmium, while lead and other PFAS were close to the null or slightly negative. SkinBloodAge showed positive associations for cadmium and selected PFAS, although cadmium remained the dominant signal.

For second-generation clocks, clearer differences were observed. PhenoAge showed positive associations with cadmium and PFNA, while other exposures remained close to the null with greater uncertainty. GrimAge showed the strongest response, with the largest positive estimates for cadmium compared to PFAS and lead. DunedinPoAm showed positive associations primarily for cadmium, with PFAS and lead remaining near zero across mixture levels.

##### Single-Variable Interaction Effects of Metals and PFAS on Epigenetic Aging

Figure 8 illustrates single-variable interaction effects of PFAS and metals on epigenetic aging, indicating whether the association between each exposure and aging changes under different mixture conditions. Interaction estimates represent the difference in effects when other exposures are fixed at higher versus lower levels (75th percentile compared with the 25th percentile).

Interaction patterns varied across outcomes but were generally weak. For first-generation clocks, most exposures showed little evidence of interaction, with estimates close to zero. HannumAge showed a modest positive interaction for cadmium, although uncertainty remained. HorvathAge and SkinBloodAge showed largely stable patterns, with minimal change in associations across mixture levels.

For second-generation measures, some variability was observed. PhenoAge showed modest positive interaction estimates for selected PFAS and lead, though with wide credible intervals. GrimAge showed limited interaction overall, with most estimates near the null and slight positive estimates for PFNA and cadmium. DunedinPoAm showed no evidence of interaction, with all estimates centered near zero.

## 4. Discussion

Our study examined the combined effects of PFAS, lead, and cadmium on multiple epigenetic aging measures in a nationally representative sample of U.S. adults aged 50 years and older. We found that cadmium showed the most consistent positive associations across epigenetic clocks, while PFAS showed more variable, clock-specific patterns. Overall, stronger associations were observed for second-generation epigenetic clocks, especially GrimAge, compared with first-generation clocks. Mixture analyses also showed that higher combined exposure levels were linked to higher DNA methylation age.

In multivariable linear regression models, cadmium remained consistently and significantly associated with all epigenetic aging measures. The strongest effects were seen for GrimAge and, to a lesser extent, PhenoAge, suggesting that cadmium may primarily influence biological aging through pathways related to physiological dysregulation rather than chronological aging. In contrast, PFAS showed less consistent patterns. PFNA was associated with GrimAge, while other PFAS showed small and variable associations, including both positive and negative directions, across the remaining clocks. Lead was also significantly associated with GrimAge. Although the effect was smaller and less consistent than that of cadmium, this finding is important because GrimAge is strongly linked to disease risk and mortality [28], suggesting that even modest elevations in lead exposure may contribute to a meaningful increase in biological aging.

Spearman correlation analyses showed strong intercorrelations among PFAS compounds, suggesting shared exposure sources and co-occurrence in the environment. In contrast, correlations between epigenetic aging measures and individual exposures were generally weak, especially for first-generation clocks. Second-generation clocks, especially GrimAge and DunedinPoAm, showed small positive relationships with cadmium and, to a lesser extent, lead, suggesting that these clocks may be more responsive to environmental exposures than first-generation measures. Although these correlations were small, they may reflect non-linear or context-dependent relationships rather than the absence of an effect. The strong intercorrelation among PFAS also suggests that evaluating exposures individually may hide joint or combined effects, supporting the use of BKMR to assess nonlinear and mixture-based relationships with epigenetic aging.

### 4.1. Cadmium as a Consistent Predictor of Increased Biological Aging

Within BKMR, cadmium showed high posterior inclusion probabilities, particularly for GrimAge and DunedinPoAm, with moderate contributions for PhenoAge and HannumAge, indicating its importance as a key driver within the exposure mixture. In univariate exposure–response functions, cadmium showed non-linear (inverted U-shaped) associations with epigenetic aging, with effects increasing at lower to moderate exposure levels and leveling off at higher levels. When we looked at pairs of exposures together, cadmium stood out for the second-generation clocks, where aging measures increased more noticeably at moderate to higher cadmium levels. This pattern likely reflects a threshold where most of the biological response occurs early on and physiological adaptation occurs at high exposure levels, suggesting that the body may deploy compensatory mechanisms at high thresholds [53]. When each exposure was examined individually within the mixture model, cadmium again showed the strongest and most consistent effects, while other pollutants were present at different levels. The interaction results suggested that cadmium’s effects might become slightly stronger when overall mixture levels were higher, but mainly, its influence appeared to be independent of other exposures. Overall exposure effect showed that higher combined exposure levels were associated with greater biological aging, with the strongest evidence for GrimAge. Consistent with our findings, Ryoo et al. reported positive associations between blood cadmium and multiple epigenetic clocks, including HannumAge, GrimAge, GrimAge 2, PhenoAge, and DNAmTL in older U.S adults, with GrimAge showing the strongest association [54]. This pattern is further supported by other studies that have reported positive associations between cadmium and epigenetic age acceleration, particularly for GrimAge [43], as well as for both GrimAge and DunedinPoAm [40,41] across individual and mixture-based analyses Cadmium has been shown to induce oxidative stress in human cells through the generation of reactive oxygen species, causing double-stranded DNA breaks and formation of telomere dysfunction-induced foci and promoting chronic inflammation [9,55], Additionally, cadmium is known to promote cellular senescence through over expression of senescence-associated secretory phenotype (SASP) factors such as IL-1α, CCL2, TGF-β via the NF-κB signaling pathway, potentially increasing epigenetic drift [56]. These mechanisms jointly explain cadmium’s association with biological aging. Cadmium’s stronger and consistent association with GrimAge reflects its broader effects on physiological health rather than changes related to chronological age. GrimAge was developed using DNA methylation surrogates of circulating proteins and mortality- related biomarkers, including plasminogen activator inhibitor-1 (PAI-1) and growth differentiation factor 15 (GDF15), which are closely linked to inflammation, vascular dysfunction, and cellular stress [28,29]. Because cadmium is known to drive these same pathways, its strong association with GrimAge may reflect this biological overlap. The positive association between cadmium and DunedinPoAm suggests that cadmium exposure may increase the pace of biological aging, consistent with DunedinPoAm’s design as a measure of the rate of physiological decline [30]. Additionally, cadmium-related biological responses may vary over time due to changes in environmental exposure and underlying physiological processes [57]; such variability may influence molecular responses and represents an important area for future research.

### 4.2. Lead and Epigenetic Aging

Lead exhibited more clock-specific and limited associations compared to cadmium. Posterior inclusion probabilities indicated that lead contributed most to the PhenoAge mixture, with a moderate PIP (0.422), while PIPs for GrimAge, first-generation clocks, and DunedinPoAm were low or near zero. This suggests that within the mixture framework, lead played a greater role in explaining variation in PhenoAge than in other epigenetic measures. Mixture analyses showed that the clearest association for lead was with GrimAge, while associations with first-generation clocks and DunedinPoAm were small or near zero. In bivariate exposure–response analyses, lead showed modest joint associations with GrimAge and, to a lesser extent, PhenoAge. Single-variable analyses also indicated that lead was primarily associated with GrimAge. Interaction effects were generally small, suggesting that lead’s influence was independent of other exposures in the mixture. Although its associations were less consistent than those observed for cadmium, the selective relationship with GrimAge is notable. GrimAge reflects inflammatory and metabolic processes linked to disease and mortality [28], which provides a plausible explanation for this. Lead has been shown to disrupt calcium signaling, promote the generation of reactive oxygen species, and interfere with endocrine regulation, all of which are involved in aging-related molecular pathways [11,36,58]. In addition, lead exposure has been shown to induce hypomethylation of the amyloid precursor protein promoter, contributing to neurotoxicity [59]. More broadly, such hypomethylation reflects broader epigenetic dysregulation that can promote cellular stress and inflammation, processes that align with the biological domains captured by GrimAge, which incorporates DNA methylation surrogates of inflammatory and mortality-related biomarkers [20,28]. Our findings are in line with prior studies. Lead exposure has been associated with higher GrimAge acceleration in a sample of 290 adults in the Detroit Neighborhood Health Study, aligning [39] and Ryoo et al. reported similar associations in older U.S. adults [54]. Additional studies have also reported consistent associations between lead and GrimAge across both linear regression and mixture-based analyses, supporting the consistency of this relationship [41]. Together, these studies strengthen the evidence that lead may contribute to biological aging pathways that influence mortality-related epigenetic clocks. However, Ryoo et al. also reported associations between lead and first-generation clocks such as HannumAge and SkinBloodAge, which we did not observe [54]. Although both studies used NHANES data, differences in findings may reflect variations in model specification, covariate adjustment, and analytic sample size due to differences in exclusions or missing data, which can influence estimated associations.

### 4.3. PFAS and Epigenetic Aging: Clock-Specific and Heterogeneous Patterns

Compared with cadmium, PFAS showed modest, outcome-specific contributions to epigenetic aging. Posterior inclusion probabilities for PFAS were generally low across all clocks except for PhenoAge, which showed a more distributed pattern. PFNA emerged as the most consistent PFAS, particularly for GrimAge and, to a lesser extent, PhenoAge, while other PFAS showed weaker associations. EPAH showed a higher contribution for SkinBloodAge, highlighting the heterogeneous and clock-specific nature of PFAS effects. In mixture analyses, most PFAS showed flat univariate exposure–response patterns, suggesting that increasing exposure did not consistently increase epigenetic aging when other exposures were held constant. Modest positive associations were observed for PFNA, particularly with GrimAge, while PFHS showed weaker and less consistent patterns. Interaction estimates were close to zero, indicating that PFAS effects were mostly independent of overall mixture levels. Bivariate exposure–response associations were mainly observed at moderate exposure levels for PFNA and PFHS with PhenoAge, suggesting possible threshold-like behavior rather than a steady increase across the full exposure range. The more consistent associations observed for PFNA may reflect its biological and physicochemical properties. PFNA has a relatively longer half-life compared to several other PFAS, which may promote accumulation and prolonged interaction with biological systems [60,61]. It has been shown to activate peroxisome proliferator-activated receptor alpha (PPARα), a regulator of lipid metabolism, inflammation, and energy balance [62]. Disruption of these pathways aligns with processes captured by second-generation clocks such as GrimAge and PhenoAge, which reflect metabolic and inflammatory function [27,28]. PFAS may influence epigenetic aging through several mechanisms. They can induce oxidative stress, generating reactive oxygen species, which can damage cellular components [37]. PFAS have been shown to impair DNA-protein interactions, reducing the ability of DNA-binding proteins to protect DNA under oxidative conditions, as demonstrated using protamine-like proteins as a model [63]. In addition, PFAS may interact with proteins involved in gene regulation, disrupting DNA-protein interactions and altering chromatin structure. These changes can affect transcriptional activity and contribute to epigenetic alterations [2].

Prior studies examining PFAS and epigenetic aging have reported mixed findings across populations and exposure contexts. Our findings extend prior research by evaluating PFAS alongside metals within a mixture framework. Some studies have observed positive associations between PFAS and epigenetic aging [64] while others have reported changes in DNA methylation without clear evidence of epigenetic age acceleration, despite very high PFAS concentrations in the exposed group [65]. In another study of older adults, PFNA has been identified as a PFAS of interest, showing consistent associations with multiple epigenetic clocks, including HorvathAge, SkinBloodAge, and PhenoAge in males [42]. This finding aligns with our results and highlights PFNA as a relevant contributor to biological aging. Differences across studies may reflect variation in age, exposure levels, and analytical approach. In our study, PFAS were evaluated within a multipollutant mixture alongside lead and cadmium, which may attenuate individual PFAS estimates compared to single-exposure analyses. Strong intercorrelations among PFAS may also make it difficult to isolate the effects of individual compounds. The higher contribution of EPAH for SkinBloodAge likely reflects relative importance within the mixture [50] rather than a stronger independent effect and may be influenced by shared exposure patterns among PFAS. Overall, these findings highlight the heterogeneous and clock-specific nature of the PFAS associations observed.

### 4.4. Differential Sensitivity of Epigenetic Clock Generations

A notable pattern across analyses was the greater responsiveness of second-generation clocks, particularly GrimAge and the third-generation measure DunedinPoAm, compared to first-generation clocks. First-generation clocks are designed to estimate chronological age [24,25,26], whereas second-generation clocks incorporate CpG sites linked to morbidity, inflammation, and mortality risk [27,28], whereas third-generation clocks capture the pace of biological aging [30]. The stronger associations observed for cadmium and, to a lesser extent, lead and PFNA, suggest that environmental toxicants may influence biological aging primarily through pathways related to physiological decline and disease risk rather than chronological aging. Because second and third-generation measures capture these processes, they appear more sensitive to environmental toxicants than first-generation clocks. This pattern is consistent with previous studies showing that second- and third-generation clocks are more responsive to environmental exposures than first-generation measures, often reflected in larger or more pronounced effect estimates [40,41,54]. This distinction highlights the importance of selecting appropriate aging metrics when evaluating the health effects of environmental exposures.

### 4.5. Study Strengths and Limitations

Our study has several notable strengths. First, we leveraged data from a nationally representative sample of older U.S. adults, enhancing the generalizability of findings to the broader population aged 50 years and older. The use of NHANES data also allowed for rigorous measurement of serum PFAS and blood metal concentrations using standardized laboratory methods, reducing exposure misclassification. Second, we evaluated multiple epigenetic aging measures spanning first, second, and third-generation clocks. This approach enabled comparison of clock-specific sensitivity to environmental toxicants and provided insight into whether exposures influence chronological aging metrics differently from mortality- and health-related aging measures. Additionally, the use of BKMR to model non-linear and interactive effects of PFAS and metal mixtures provides a comprehensive understanding of exposure–response relationships. Finally, the use of multiple imputation to address missing data improved statistical efficiency and reduced potential bias associated with incomplete exposure and covariate information. Our study is not without limitations. First, the cross-sectional design of NHANES precludes causal inference and limits the ability to establish temporal relationships between exposure and epigenetic aging. Second, exposure biomarkers were measured at a single time point. While cadmium and lead may reflect cumulative body burden to some extent, PFAS concentrations may vary over time depending on exposure patterns and elimination kinetics. Therefore, single measurements may not fully capture long-term exposure history relevant to biological aging processes. Third, although models were adjusted for key demographic and socioeconomic covariates, residual confounding by unmeasured factors—such as diet, occupational exposures, smoking, or other environmental chemicals—remains possible. We also ran a large number of models, which increases the potential for false positives. We conducted multiple statistical tests across exposures and outcomes without applying a formal multiple comparison correction. Given the exploratory nature of the analysis, findings were interpreted cautiously, with greater emphasis placed on consistency, biological plausibility, and concordance across analytic approaches rather than isolated *p*-values. As a result, some findings may reflect chance associations. Finally, a methodological consideration relates to the use of raw epigenetic clock values rather than residual-based epigenetic age acceleration (EAA) measures for analyses. Although models were adjusted for chronological age, the first-generation clocks are trained to predict chronological age and therefore inherently contain an age-related signal. As a result, effect estimates may not be directly equivalent to residual-derived EAA metrics. However, this approach allows for direct interpretation of biological age differences at a given chronological age. Findings should therefore be interpreted as associations with epigenetic age conditional on chronological age rather than pure acceleration measures. Future work using harmonized residual-based EAA approaches may further clarify these relationships.

## 5. Conclusions

This study highlights the joint effects of PFAS, cadmium, and lead on epigenetic aging in this nationally representative sample of U.S. adults aged 50 years and older. Across analytic approaches, cadmium emerged as the most consistent and influential exposure, showing positive associations across first, second, and third-generation aging measures. Associations were stronger for second- and third-generation clocks, particularly GrimAge and DunedinPoAm, while first-generation clocks showed more modest responsiveness. PFAS exhibited heterogeneous, clock-specific patterns, with PFNA demonstrating the most consistent associations among PFAS compounds. Lead was selectively associated with GrimAge. Mixture analyses revealed nonlinear exposure–response relationships and highlighted cadmium as a dominant contributor within the exposure mixture. Higher combined exposure levels were associated with increased epigenetic aging, particularly for GrimAge. These findings suggest that cumulative exposure burden contributes to biological aging beyond individual exposure effects and underscore the importance of mixture-based approaches in environmental health research. Interaction effects were generally limited, suggesting that most associations remained relatively unchanged across mixture contexts. Collectively, these findings suggest that co-occurring environmental exposures are associated with differences in epigenetic aging measures in this population. These findings have important implications for public health. The consistent association between cadmium and epigenetic aging suggests that reducing exposure to this toxicant may be particularly relevant for mitigating biological aging processes. Given the widespread presence of PFAS and metals in the environment, efforts to reduce cumulative exposure burden may have meaningful impacts on aging-related health outcomes, especially in populations with higher environmental risk. Longitudinal research is needed to clarify temporal relationships and determine whether reductions in exposure correspond to measurable changes in biological aging measures.

## Figures and Tables

**Table 1:** Survey-weighted descriptive statistics of continuous variables.

Variable	Mean (SE)
Age (years)	63.2 (0.34)
BMI (kg/m^2^)	28.8 (0.39)
Lead	2.71 (0.12)
Cadmium	0.70 (0.04)
EPAH	0.81 (0.08)
MPAH	0.90 (0.10)
PFHS	2.96 (0.22)
PFNA	0.81 (0.07)
PFOA	6.14 (0.52)
PFOS	38.25 (2.63)
PFSA	0.50 (0.03)

Values are survey-weighted means with standard errors. Estimates account for the complex NHANES sampling design. Blood lead and cadmium concentrations are reported in µg/dL; serum PFAS concentrations in ng/mL.

**Table 2:** Survey-weighted distribution of categorical variables.

Variable	*N*	Percentage (%)
**Sex**		
Male	569	45.0
Female	550	55.0
**Race/Ethnicity**		
Mexican American	393	4.3
Other Hispanic	90	7.4
Non-Hispanic White	375	75.9
Non-Hispanic Black	225	8.9
Other Race-including multi-racial	36	3.5
**Education**		
Less than 9th grade	370	12.0
9–11th grade (no diploma)	239	20.7
High school graduate/GED	210	27.3
Some college or AA degree	174	22.2
College graduate or above	126	17.9
**Income**		
<USD 20,000	355	28.3
USD 20,000–USD 34,999	209	21.8
USD 35,000–USD 54,999	144	19.8
>USD 55,000	181	30.2

Values are presented as unweighted counts and weighted percentages to account for the complex NHANES sampling design.

**Table 3:** Linear Regression Results for Epigenetic Clocks.

**Exposure**	**β (Estimate)**	**Std. Error**	***p*-Value**
Lead	0.013	0.055	0.812
Cadmium	1.043	0.421	0.013
EPAH	0.136	0.229	0.555
MPAH	0.637	0.443	0.159
PFHS	0.052	0.130	0.690
PFNA	0.017	0.634	0.979
PFOA	0.019	0.064	0.77
PFOS	0.004	0.024	0.873
PFSA	−0.832	0.689	0.235
**(A) HannumAge**			
**Exposure**	**β (Estimate)**	**Std. Error**	***p*-Value**
Lead	0.013	0.056	0.811
Cadmium	0.977	0.415	0.019
EPAH	0.025	0.189	0.896
MPAH	0.246	0.501	0.626
PFHS	0.111	0.149	0.460
PFNA	1.101	0.577	0.064
PFOA	−0.035	0.062	0.57
PFOS	−0.029	0.024	0.247
PFSA	−0.007	0.570	0.990
**(B) HorvathAge**			
**Exposure**	**β (Estimate)**	**Std. Error**	***p*-Value**
Lead	0.043	0.050	0.390
Cadmium	0.823	0.375	0.028
EPAH	−0.150	0.161	0.356
MPAH	0.199	0.391	0.613
PFHS	0.021	0.119	0.860
PFNA	0.376	0.503	0.459
PFOA	0.021	0.062	0.73
PFOS	0.009	0.022	0.680
PFSA	−0.372	0.492	0.452
**(C) SkinBloodAge**			
**Exposure**	**β (Estimate)**	**Std. Error**	***p*-Value**
Lead	0.061	0.068	0.370
Cadmium	2.467	0.516	<0.001
EPAH	−0.052	0.293	0.860
MPAH	0.133	0.503	0.793
PFHS	0.096	0.176	0.589
PFNA	0.729	0.637	0.258
PFOA	−0.023	0.081	0.78
PFOS	−0.018	0.028	0.531
PFSA	−0.366	0.701	0.603
**(D) PhenoAge**			
**Exposure**	**β (Estimate)**	**Std. Error**	***p*-Value**
Lead	0.116	0.041	0.005
Cadmium	5.385	0.306	<0.001
EPAH	−0.090	0.156	0.567
MPAH	0.483	0.319	0.138
PFHS	0.027	0.113	0.812
PFNA	1.159	0.480	0.021
PFOA	0.053	0.046	0.26
PFOS	−0.006	0.019	0.738
PFSA	−0.236	0.515	0.649
**(E) GrimAge**			
**Exposure**	**β (Estimate)**	**Std. Error**	***p*-Value**
Lead	0.0016	0.0009	0.077
Cadmium	0.0985	0.0067	<0.001
EPAH	0.0012	0.0028	0.678
MPAH	0.0038	0.0080	0.637
PFHS	0.0012	0.0025	0.647
PFNA	0.0115	0.0089	0.203
PFOA	0.001	0.001	0.32
PFOS	−0.0001	0.0004	0.693
PFSA	−0.0077	0.0095	0.425
**(F) DunedinPoAm**			

Values are regression coefficients (β) with standard errors (SE) and *p*-values from linear regression models adjusted for age, sex, race/ethnicity, education, income, and BMI. *p*-value is significant at *p* < 0.05.

**Table 4:** Posterior Inclusion Probabilities by Epigenetic Clock.

Exposure	HannumAge	HorvathAge	SkinBloodAge	PhenoAge	GrimAge	DunedinPoAm
Lead	0.000	0.009	0.043	0.422	0.050	0.000
Cadmium	0.560	0.124	0.280	0.832	1.000	1.000
EPAH	0.000	0.005	0.408	0.466	0.018	0.000
MPAH	0.012	0.013	0.152	0.348	0.028	0.000
PFHS	0.002	0.016	0.070	0.494	0.025	0.000
PFNA	0.006	0.172	0.143	0.503	0.062	0.000
PFOA	0.024	0.046	0.136	0.457	0.000	0.000
PFOS	0.004	0.079	0.174	0.384	0.002	0.000
PFSA	0.048	0.043	0.199	0.466	0.007	0.000

## Data Availability

The original data presented in this study are openly available in the National Health and Nutrition Examination Survey (NHANES) repository at https://wwwn.cdc.gov/nchs/nhanes/continuousnhanes/overview.aspx?BeginYear=2001 (accessed on 12 March 2026).
